# Complexity at a Humid Interface: Throwing Fresh Light on Atmospheric Corrosion

**DOI:** 10.1021/acsami.4c21013

**Published:** 2025-04-23

**Authors:** Michael Dowhyj, Kiran Kousar, Francis P. Lydiatt, Dimitri Chekulaev, Monika S. Walczak, Robert Temperton, James N.O’Shea, W. Stephen Walters, Andrew G. Thomas, Robert Lindsay

**Affiliations:** † Corrosion@Manchester, Department of Materials, The University of Manchester, Manchester, Manchester M13 9PL, U.K.; ‡ Photon Science Institute, The University of Manchester, Manchester M13 9PL, U.K.; § School of Physics and Astronomy, University of Nottingham, Nottingham NG7 2RD, U.K.; ∥ UK National Nuclear Laboratory, Culham Science Centre Abingdon, Oxfordshire OX14 3DB, U.K.; ⊥ Department of Materials, The University of Manchester, Manchester M13 9PL, U.K.

**Keywords:** atmospheric corrosion, relative humidity, adventitious carbon, capillary condensation, in situ measurements, X-ray photoelectron spectroscopy, vibrational sum frequency spectroscopy

## Abstract

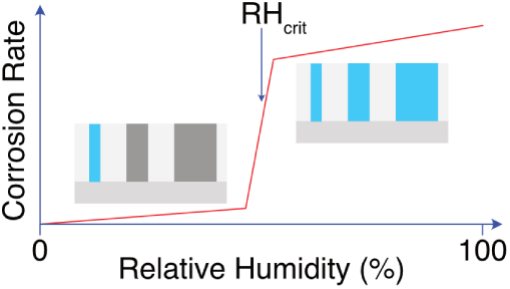

Atmospheric corrosion of metals arising from exposure to water vapor is a pervasive problem across a wide range of practical scenarios, including nuclear material storage and historical artifact conservation. Frequently, it is hypothesized that this phenomenon becomes an issue once the number of monolayers of water growing atop a substrate is sufficient to facilitate corrosion chemistry, but supporting evidence remains scarce. We apply both near ambient pressure X-ray photoelectron spectroscopy and vibrational sum frequency spectroscopy to further elucidate the interaction of water vapor with zinc, a common engineering substrate for corrosion protection applications. Data acquired as a function of relative humidity indicate that water sorption is much more complex than expected, involving micropore filling and capillary condensation in the adventitious carbon layer covering the zinc surface. These results suggest that current mechanistic models for atmospheric corrosion, as well as other interfacial phenomena occurring in humid environments, require extensive revision and should embrace explicit consideration of the role of surface carbon contamination.

## Introduction

Wetting of solid surfaces in humid environments drives various real-world physicochemical processes, ranging from the reduction of friction to substrate degradation. Concerning the latter, a topic of particular interest is deterioration of metallic substrates through atmospheric corrosion, as it can result in the loss of both cosmetic appearance and structural integrity.^1^ In the 1930s, Vernon undertook seminal work in this area, establishing that corrosion can become significant prior to the surface condensation of bulk liquid water; i.e., the relative humidity (RH) of the environment can be lower than 100%.^2^ Commonly, this behavior is proposed to result from the accumulation of adsorbed monolayers (ML) of water atop a substrate, which become sufficient in number to support corrosion chemistry once a critical RH (RH_crit_) is exceeded.^3,4^ Experimental data supporting this hypothesis do exist,^5−8^ but nanoscale details of the water layers at pertinent RH values remain scarce, impeding full mechanistic understanding of atmospheric corrosion. In this study, we deliver fresh insight into this phenomenon through the application of two *in situ* surface spectroscopies, namely near ambient pressure X-ray photoelectron spectroscopy (NAP-XPS)^9^ and vibrational sum frequency spectroscopy (VSFS).^10^

NAP-XPS has already been widely exploited for studying water adsorption, e.g., refs.^11−26^. On oxidized metallic surfaces, data demonstrate that water often undergoes surface/defect-mediated dissociation at ultralow values of RH.^11,13,15,17,21,22,24,25^ This reaction results in chemisorbed surface hydroxyl (OH_ads_) groups, which can facilitate the subsequent binding of molecular water. For example, NAP-XPS spectra, acquired from a single crystal Fe_2_O_3_(0001) surface for 0% ≤ RH ≤ 34%, indicate that surface hydroxylation begins at RH ∼ 1 × 10^–7^%.^13^ Molecular water starts to adsorb once RH ≳ 2 × 10^–5^%, with greater than monolayer coverage being observed at RH = 3.4 × 10^1^% (34%). These results are clearly of import, but they do not divulge the nature of the water layer at RH values coincident with the onset of significant atmospheric corrosion, i.e., RH_crit_ ∼ 70%–80% on a deposit-free surface;^27^ most analogous NAP-XPS studies also focus on RH values below those of most interest for atmospheric corrosion.^15,17,21,22,24,25^

Here, the deficiency of previous NAP-XPS studies apropos atmospheric corrosion is addressed through acquiring data over a broad range of RH. Specifically, we focus on the interaction between water vapor (0% ≲ RH ≲ 100%) and the oxidized surface of polycrystalline zinc (RH_crit_ ∼ 70%);^27^ the latter is an important material for atmospheric corrosion control.^28^ VSFS measurements, which reveal the vibrational signature of the humid interface, provide complementary insight into the structure of the sorbed water as a function of RH. By combining evidence emerging from both spectroscopic measurements, it is concluded that water does not simply grow atop a sharply terminated zinc substrate but instead accumulates through micropore filling/capillary condensation in the ubiquitous layer of adventitious carbon covering the surface.

## Results and Discussion

**Figure 1:**
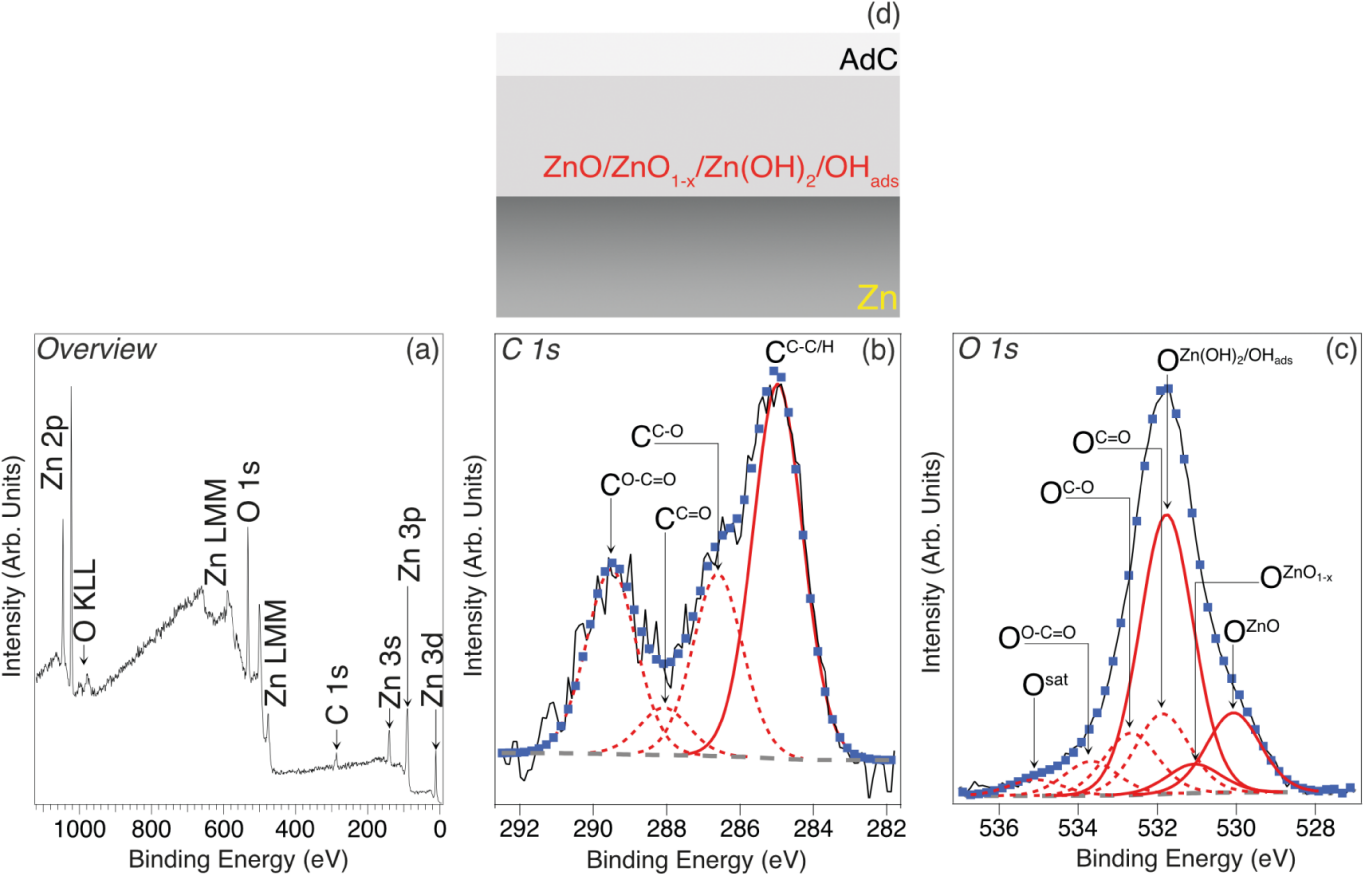
(a) Overview, (b) C 1s, and (c) O 1s XPS spectra (hν = 1486.6 eV, θ_E_ = 0°) acquired from a polished Zn substrate that has undergone UV-ozone exposure prior to insertion into the NAP-XPS instrument. Blue markers show best fits to the experimental C 1s and O 1s spectra (black lines), using GL(30) (red solid/broken lines) and Shirley-type (broken gray lines) functions.^31^ The peak labels are explained in the main text. (d) Illustration of surface termination, as concluded from the XPS data, i.e., the surface of the metallic Zn substrate is oxidized to form a layer consisting of ZnO, ZnO_1–*x*_, Zn(OH)_2_, and OH_ads_, which is covered by AdC. It should be noted that although metallic Zn is included in this schematic cross section, the Zn L_3_M_45_M_45_ Auger peak profile (see Figure S1) indicates that the thickness of oxidized layer is such that the signal from the underlying metallic Zn is minimal.^29^

Figure 1a shows an overview XPS spectrum of the as-prepared polycrystalline Zn prior to exposure to water vapor, i.e., RH = 0%. Features assigned to various Zn and O core level XPS peaks (Zn 2p/3s/3p/3d and O 1s) are indicated, along with associated Auger transitions (Zn LMM and O KLL). As expected for a Zn sample exposed to air, the features are consistent with the surface being terminated by a film of oxidized Zn;^29^ the profile of the Zn L_3_M_45_M_45_ Auger feature supports the presence of such a surface film (see Figure S1).^29^

Besides the Zn- and O-derived signals in Figure 1a, there is a peak assigned to photoemission from the C 1s core level. This feature can be attributed to surface-adsorbed adventitious carbon (AdC), which is invariably present on inorganic surfaces exposed to atmosphere;^30^ the initial amount of such carbon has been reduced through subjecting the sample to UV-ozone treatment immediately prior to insertion into the NAP-XPS instrument. A higher-resolution spectrum of this core level is displayed in Figure 1b. It has been fitted with 4 Gaussian–Lorentzian (GL) line shape functions to describe photoemission from specific C environments and a Shirley-type function to account for the secondary electron background;^31^ the best-fit binding energy (BE) and full width at half maximum (fwhm) for each of these components are listed in Table S1. The peak labeled C^C–C/H^, which was set to a binding energy (BE) of 285.0 eV during calibration, is assigned to C atoms bonded to C and/or H in surface-bound AdC.^30^ C^C–O^, C^C=O^, and C^O–C=O^ are attributed to C atoms bonded to one or more O atoms in the same layer (see Table S1 for more details).^30^

Further details of the nature of the as-prepared Zn surface can be derived from the higher resolution, O 1s core-level XPS data displayed in Figure 1c. To account for contributions to this spectrum from the O atoms in both the oxidized surface Zn film and AdC, a total of 7 GL line shape functions and a Shirley-type background function have been employed for fitting (see Table S1 for the best-fit BE and fwhm values; the latter were constrained to all have the same value during fitting). Following refs.^32,33^ O atoms in various chemical environments (i.e., different O to C bonding configurations) in the adventitious AdC layer are represented by three peaks, i.e., O^C=O^, O^C–O^, and O^O–C=O^ (see Table S1 for more details). Another three O 1s peaks at BEs of 530.0 eV (O^ZnO^), 531.0 eV (), and 531.7 eV () are allocated to the oxidized Zn layer, being assigned to the presence of stoichiometric ZnO,^34−37^ nonstoichiometric ZnO_1–*x*_,^38^ and Zn(OH)_2_ plus surface-bound hydroxyls (OH_ads_),^21,34,37,39,40^ respectively. The origin of the final component (O^sat^) in Figure 1c is less certain, but we speculate it may be a so-called shakeup satellite associated with one of the oxidized zinc phases.^22^

Figure 1d summarizes the surface structure/chemistry of the as-prepared Zn sample as concluded from the XPS data in Figure 1a–c, i.e., the surface of the metallic Zn substrate is oxidized to form a layer comprising ZnO, ZnO_1–*x*_, Zn(OH)_2_, and OH_ads_, which is covered by AdC. This termination is used as a baseline for elucidating the impact of varying RH.

**Figure 2:**
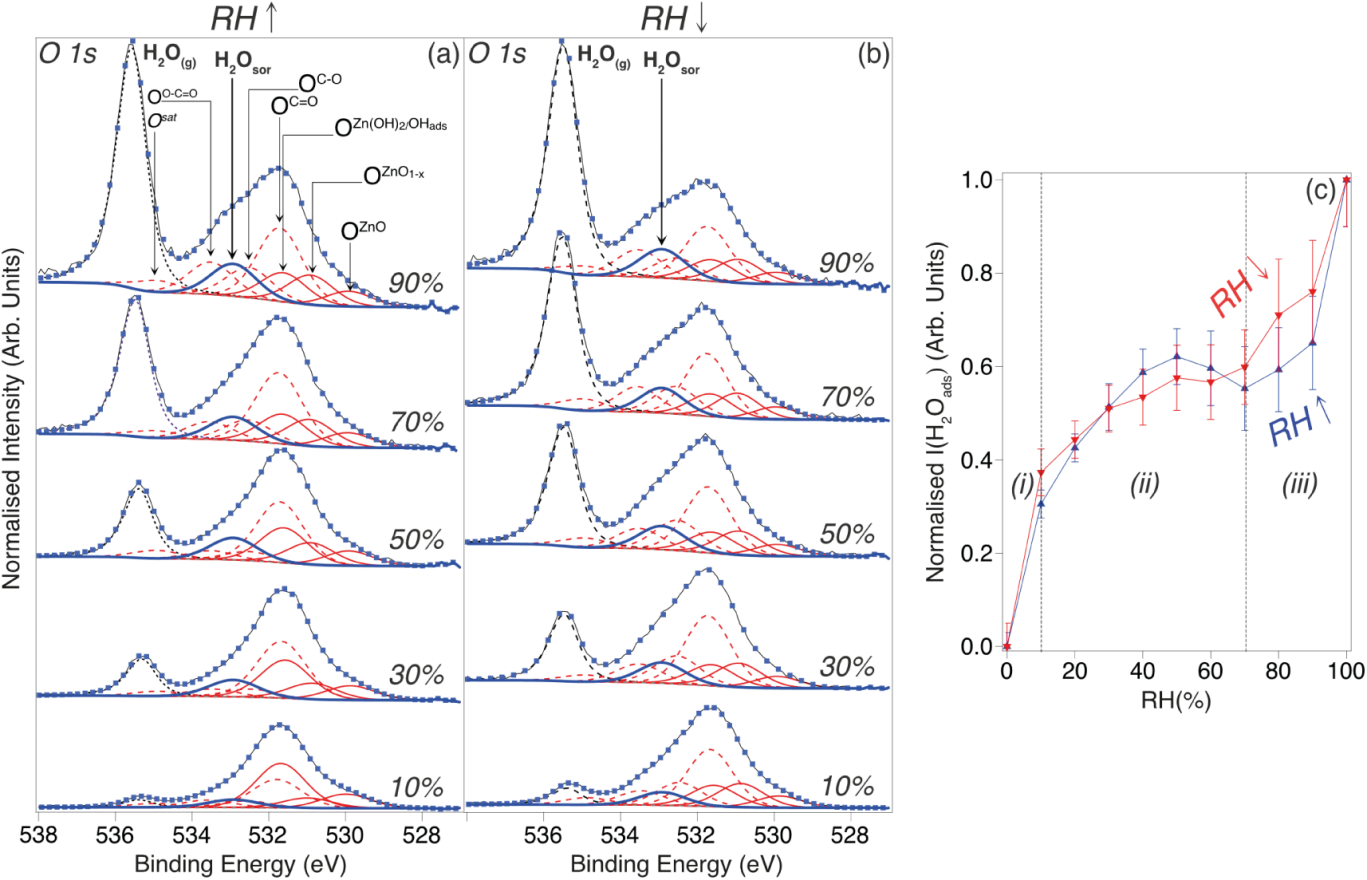
O 1s XPS spectra (hν = 1486.6 eV, θ_E_ = 0°) acquired from polished/UV-ozone exposed Zn as a function of (a) increasing RH and (b) decreasing RH. Blue markers show best fits to the experimental data (black lines), using GL(30) (red solid/broken lines) line shape functions for all of the components except for gas phase water (H_2_O_(g)_), where an asymmetric Lorentzian (LA) line shape convoluted with a Gaussian function (black broken line) was employed;^49^ Shirley-type (broken gray lines) functions were used to describe the secondary electron background.^31^ Spectra have been normalized to the sum of the signals assigned to O^ZnO^, , , and O^sat^. (c) Plot of O 1s intensity of sorbed water (I(H_2_O_sor_)) as a function of RH. Intensity has been normalized to the sum of the O 1s signals assigned to O^ZnO^, , , O^C=O^, O^C–O^, O^O–C^=^O^, and O^sat^, and then further normalized so that the intensity is 1.0 at RH = 100%; error bars have been determined from an estimate of the uncertainty in the best fits. Regions *(i)*, *(ii)*, and *(iii)*, separated by vertical gray dashed lines, are discussed in the text.

Selected O 1s XPS spectra, which have been acquired as a function of increasing and decreasing RH, are shown in Figure 2a,b, respectively. Best fits to these data are also indicated, which have been achieved through the addition of 2 further core-level line shape functions to the 7 used for the RH = 0% data (Figure 1c), as well as allowing the intensity of other components to vary (see Table S2 for best-fit peak areas); the two additional core-level functions were the minimum number of extra components required to fit the O 1s data as a function of RH. We note that during fitting the BE of the 7 original components was constrained to be essentially the same (±0.1 eV) as those used for the fitting displayed in Figure 1, and a single fwhm value was employed.

The first of the two added O 1s components, located at BE ∼ 535.5 eV, is a result of photoemission from gas-phase water (H_2_O_(g)_) in the measurement chamber;^13^ this feature increases/decreases with RH, as the partial pressure of H_2_O(g) is systematically changed (0 mbar–7 mbar) to deliver the required RH range (0%–100%). The other additional feature (BE ∼ 532.9 eV), which similarly increases/decreases with RH, is attributed to surface-sorbed water (H_2_O_sor_).^11,13,15,20,22,25,26^ The O^C=O^, O^C–O^, and O^O–C=O^ peaks also vary in intensity, which we propose to be a result of the quantity of surface AdC increasing upon exposure to the NAP environment, as discussed in other studies (e.g., displacement of AdC precursors from the chamber walls).^11,13,15,20,22,25^ Corresponding C 1s data and their best fits in Figure S2a,b validate this assertion, i.e., they show a similar trend versus RH, as verified by the plot in Figure S2c, where the summed intensity of O^C=O^ + O^C–O^ + O^O–C=O^ is compared to that of C atoms bonded to one or more O in AdC (i.e., C^C–O^ + C^C=O^ + C^O–C=O^). Another variation of note in the O 1s signal in Figure 2 is the diminution of the  peak, and the concurrent gain in  intensity, which can be explained by the vulnerability of Zn(OH)_2_ to X-ray-induced damage;^35^ we stress that there was no evidence of similar damage for sorbed H_2_O, which is likely a result of having a plentiful supply of H_2_O in the vapor phase that can replace any H_2_O desorbed by the X-ray beam.

In sharp contrast to the O 1s XPS signal, the spectral profile of Zn 2p XPS data recorded as a function of RH remains essentially constant throughout the measurements. Figure S3 compares two Zn 2p spectra, one acquired at RH = 10% as RH is increased in steps (solid red line) and the other at the same RH but for decreasing RH (broken blue line), i.e., spectra acquired toward the beginning and end of the NAP-XPS measurements, respectively. Consequently, the Zn 2p spectra provide no insight into the evolution of the Zn substrate. Consistent with the Zn L_3_M_45_M_45_ Auger feature (Figure S1), the BE of the Zn 2p_3/2_ peak (1021.8 eV) and the profile of the spectra correspond to an oxidized Zn surface,^29^ with no significant contribution from the underlying Zn metal.

Figure 2c shows the normalized intensity of the O 1s peak assigned to H_2_O_sor_ (I(H_2_O_sor_)) as a function of RH. The data plotted for increasing RH are almost coincident with those for decreasing RH, suggesting that water sorption/desorption with increasing/decreasing RH is largely reversible. For a more quantitative insight, the coverage of H_2_O_sor_ has been estimated from the relative intensities of XPS peaks, under the assumption that H_2_O_sor_ uptake occurs layer-by-layer atop the AdC layer, as depicted in Figure S4. At the maximum RH probed by NAP-XPS (∼100%), the coverage was found to be ∼2.1 ML; details of the coverage calculation are provided in Supporting Information.

According to previous work on surface carbonation in humidified CO_2_,^41^ ∼2.1 ML (∼0.7 nm) of H_2_O_sor_ may be close to the threshold for the onset of key corrosion processes, such as ionic transport and solvation of dissolving metal ions. Consequently, the water layer at RH = 100% may be of sufficient depth to enable atmospheric corrosion, but at lower RH values it will almost certainly be too thin, e.g., the coverage is estimated to be ∼1.3 ML (∼0.4 nm) at RH = 70%, which has been reported to be RH_crit_ for atmospheric corrosion of zinc.^27^ Consequently, we argue that layer-by-layer uptake of H_2_O_sor_ on top of AdC is not an appropriate description of water sorption for this system. Moreover, there is a lack of direct contact between H_2_O_sor_ and the zinc oxide/hydroxide-terminated substrate in this model, which is an essential prerequisite for the occurrence of atmospheric corrosion.

**Figure 3:**
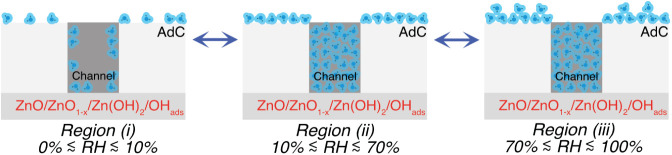
Cartoon showing the likely primary (reversible) sorption processes for H_2_O_sor_ (blue) as a function of RH, i.e., *Region (i)* (0% ≲ RH ≲ 10%): lateral monolayer growth across the substrate surface; *Region (ii)* (10% ≲ RH ≲ 70%): micropore filling/capillary condensation of channels running through the AdC; *Region (iii)* (70% ≲ RH ≲ 100%): further adsorption atop the AdC layer. It should be noted that a simple straight geometry is used to represent a channel through the AdC layer, although the real-world geometry is almost certainly more complex.

To resolve the issue of H_2_O_sor_/Zn oxide/hydroxide interaction, the AdC must allow water to access the underlying substrate, e.g., H_2_O_sor_ fills channels running through this layer. Such permeability is consistent with the I(H_2_O_sor_) versus RH data in Figure 2c, as the profile approximates a Type-II adsorption isotherm for H_2_O_sor_ uptake on porous carbonaceous substrates; liquid water can exist in the voids in such a material at RH < 100% through micropore filling/capillary condensation.^42,^^43^ Based on this assertion, Figure 3 depicts the primary (reversible) H_2_O_sor_ sorption processes proposed to occur as a function of RH, i.e., *Region (i)* (0% ≲ RH ≲ 10%): lateral monolayer growth across the substrate surface; *Region (ii)* (10% ≲ RH ≲ 70%): micropore filling/capillary condensation; *Region (iii)* (70% ≲ RH ≲ 100%): further adsorption atop the AdC layer;^43^ we want to emphasize that although a simple straight geometry is used to represent a channel through the AdC layer, the real-world geometry is almost certainly more complex. The three regions ((*i)*, *(ii),* and *(iii)*) are indicated by vertical gray dashed lines in Figure 2c.

**Figure 4:**
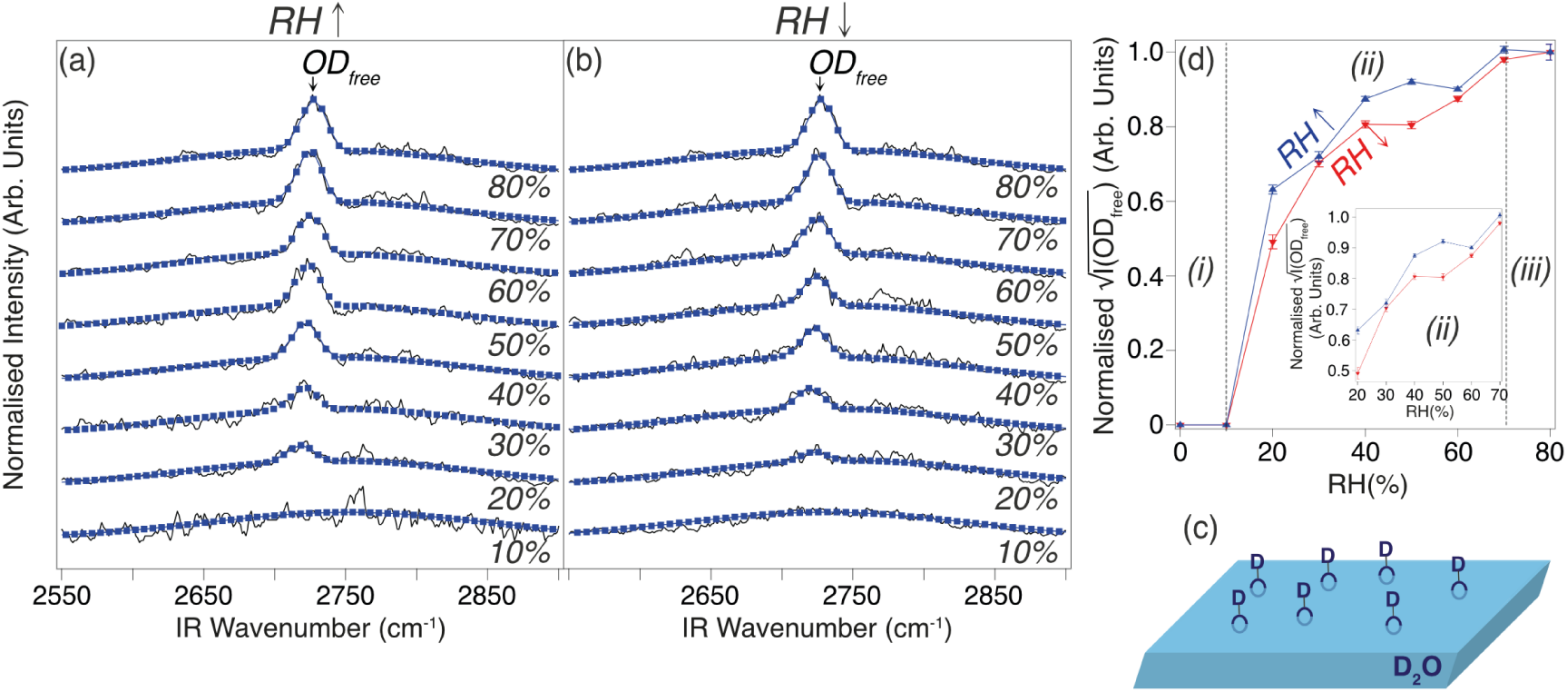
VSFS spectra (OD_free_ stretch region) acquired from polished/UV-ozone exposed Zn as a function of (a) increasing RH and (b) decreasing RH (D_2_O was used instead of H_2_O). Blue markers show best fits to the experimental data (black lines). Spectra have been normalized to the nonresonant background signal. All data were acquired with MIR pulses centered at ∼2750 cm^-1^, using a *ppp* polarization combination and τ ∼ 730 fs. (c) Cartoon showing non-hydrogen bonded OD groups (OD_free_) protruding out from liquid D_2_O. (d) Plot of the square root of the intensity of OD_free_ resonance (I(OD_free_)^0.5^) as a function of RH, which has been normalized so that it is equal to 1 at RH = 80%; error bars have been determined from an estimate of the uncertainty in the best fit. It should be noted that for visualization purposes the red/blue lines have been extended to RH = 0%, although the first experimental data points (indicated by red/blue triangles) are located at RH = 10%. *Regions (i)*, *(ii)*, and *(iii)*, separated by vertical gray dashed lines, are discussed in the text. Inset plot focuses on *Region (ii)*.

Corresponding VSFS spectra, acquired from a polycrystalline Zn sample as a function of increasing and decreasing RH (10% ≲ RH ≲ 80%), replacing H_2_O with D_2_O, are shown in Figure 4a,b, respectively. A relatively sharp feature, located at ∼2726 cm^–1^, is observed to vary in intensity with RH. From previous work, this resonance can be assigned to the so-called free OD stretch (OD_free_) of D_2_O molecules at the liquid/vapor interface,^44^ i.e., it arises from excitation of protruding (non-hydrogen bonded) OD groups, as illustrated in Figure 4c; the ∼10 cm^–1^ red shift for this feature between ref.^44^ and our data most likely arises from uncertainties in calibration and peak position identification, rather than having a significant physical origin. Best fits to the VSFS spectra, using a combination of a Voigt line-shape function for the OD_free_ resonance and a Gaussian line-shape function for the nonresonant background, are also shown in Figure 4a,b. Details of the fitting approach and best-fit parameters (Table S5) are provided in the Supporting Information.

The intensity of the OD_free_ resonance (I(OD_free_)) is expected to vary quadratically with the number of OD_free_ groups (N(OD_free_)), assuming a constant molecular orientation, i.e., I(OD_free_)^0.5^ ∝ N(OD_free_).^45^ On this basis, I(OD_free_)^0.5^ is plotted as a function of increasing/decreasing RH in Figure 4d. Focusing on *Region (i)* (0% ≲ RH ≲ 10%), despite NAP-XPS spectra (Figure 2) indicating a significant change in H_2_O_sor_ signal, which is suggested to arise from variation in first-layer H_2_O coverage, there is no apparent OD_free_ signal at RH = 10%. Consistent with the conclusions of some previous studies (see, for example ref.^46^) a likely explanation is that in the (sub)monolayer regime the D_2_O (or H_2_O) forms a rather rigid ice-like structure with extensive hydrogen bonding in *Region (i)*, resulting in few free OD species. Another related point to address is that, in contrast to the current data, Hedberg et al.^44^ reported a signal from surface-bound hydroxyl groups (OD/OH) in their VSFS data acquired upon returning to RH = 0%. This discrepancy may be a result of differences in surface preparation (e.g., Hedberg et al. did not apply a UV-ozone cleaning step) or other experimental procedures, resulting in less extensive surface hydroxylation in the current work.

Throughout *Region (ii)* (10% ≲ RH ≲ 70%), I(OD_free_)^0.5^ increases/decreases in a near reversible manner. There is a step-up in intensity between RH = 10% and RH = 20%, which we conclude is due to the onset of micropore filling/capillary condensation. Subsequently, there is a reasonably linear trend for RH = 20%–70%, as highlighted by the inset in Figure 4d. Presuming that N(OD_free_) varies linearly with the surface area of liquid D_2_O, it can be deduced that the fraction of the substrate covered by liquid D_2_O at the humid Zn interface steadily increases/decreases with RH. Building on the water sorption description in Figure 3, this behavior can be attributed to a distribution of different channel dimensions in the AdC layer, as the RH required for micropore filling/capillary condensation is dependent on their size;^42^ if there were a single channel size in the AdC layer, then the I(OD_free_)^0.5^ versus RH plot in Figure 4d would be expected to display a step-like profile.

**Figure 5:**
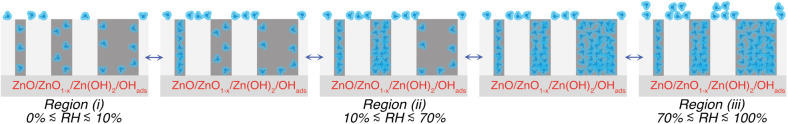
Cartoon showing the likely primary (reversible) sorption processes for H_2_O_sor_ (blue) as a function of RH, including the impact of variable channel size, i.e., *Region (i)* (0% ≲ RH ≲ 10%): lateral monolayer growth across the substrate surface; *Region (ii)* (10% ≲ RH ≲ 70%): size dependent micropore filling/capillary condensation, where larger channels become occupied at higher values of RH; *Region (iii)* (70% ≲ RH ≲ 100%): further adsorption atop the AdC layer. It should be noted that a simple straight geometry is used to represent a channel through the AdC layer, although the real-world geometry is almost certainly more complex.

Figure 5 visually conveys the impact of variable channel size on water sorption; i.e., increasingly large channels become filled as RH increases (10% ≲ RH ≲ 70%). A key question emerging from this schematic is *Despite the onset of micropore filling/capillary condensation at RH ∼ 10%, why does* ref.^27^*report RH*_*crit*_*∼70%?* Adhering to Occam’s razor, one plausible option is that channels need to be of sufficient diameter, to facilitate corrosion chemistry processes, i.e., there is a critical channel size (Ch_crit_) for the onset of significant atmospheric corrosion. On the basis of ref.^47^ we estimate Ch_crit_ to be at least 1.4 nm; the thickness of the AdC layer at RH = 100% was determined to be ∼2 nm with the XPS coverage equations described in Supporting Information. We note that this hypothesis requires validation through further experimental work and/or modeling, e.g., the application of *state-of-the-art* density functional theory (DFT) approaches, such as those recently reviewed in ref.^48^ to gain atomic-scale insight into the viability of corrosion processes as a function of liquid volume at the nanoscale. If substantiated, there is the intriguing prospect of pursuing strategies to engineer AdC layers (e.g., through chemical treatment) to provide substrate protection against atmospheric corrosion under damp conditions through limiting their channel size.

Beyond providing an alternative explanation for the origin of RH_crit_ more than 90 years after the seminal work on atmospheric corrosion by Vernon,^2^ this study has broader significance. For any researcher interested in understanding phenomena occurring at fluid/solid interfaces, the current results suggest that they should consider that AdC may play a key role and so should conduct appropriate experiments to test this possibility. Moreover, practitioners of XPS/NAP-XPS should be aware of the potential contribution of AdC to their C 1s and O 1s spectra. As discussed previously,^22^ ignoring AdC may lead to misinterpretation of data, including overestimating surface coverage.

## Conclusions

In summary, NAP-XPS and VSFS have been employed to provide fresh insight into RH_crit_ for the onset of appreciable atmospheric corrosion through examining the interaction of water vapor with zinc. XPS data demonstrate that prior to water exposure the zinc substrate is terminated by a layer of zinc oxide/hydroxide topped by AdC; formation of the latter layer is essentially inevitable upon exposure to the atmosphere. In addition, O 1s XPS core-level spectra acquired as a function of RH indicate that the quantity of surface-sorbed water varies with this parameter, but a simple layer-by-layer growth model is not an appropriate description. Instead, it is concluded that water sorption occurs through micropore filling/capillary condensation of channels in the AdC. VSFS spectra of the OD_free_ stretch region are consistent with this water uptake model, and the variation in the OD_free_ signal intensity with RH suggests a distribution of channel sizes. Consequently, it is argued that RH_crit_ coincides with the filling of channels in the AdC layer that are large enough to facilitate corrosion chemistry. Besides providing a step change in our understanding of atmospheric corrosion, this study suggests that the role of AdC should not be overlooked as a potential key factor in other interfacial phenomena.

## Methods

Circular discs (∼5 mm thickness) cut from a rod (12 mm diameter) of polycrystalline Zn, purchased from Goodfellow (Purity: 99.9%), were employed as samples for both NAP-XPS and VSFS. Once cut, a flat face of each Zn sample was ground with a series of SiC papers, and polished with diamond paste (down to 1 μm) until a mirror-finish was obtained; we note that the surface also displayed a mirror-finish following NAP-XPS/VSFS measurements as a function of RH. Subsequently, samples were degreased by successive sonication in acetone, ethanol, and deionized water, and then blown dry. Finally, just prior to NAP-XPS/VSFS measurements, samples were inserted into a UV-ozone cleaner (Novascan) to reduce surface-adhered adventitious carbon deposits.

NAP-XPS experiments were undertaken with a SPECS Devi-Sim instrument consisting of an ultrahigh vacuum preparation/analysis chamber and a NAP cell equipped with a Peltier thermoelectric sample heater/cooler. Prior to attachment to this Peltier element, the Zn sample was mounted onto a molybdenum plate and spot-welded to a thermocouple for temperature monitoring. During NAP-XPS data acquisition, the cell was secured onto the entrance aperture of a PHOBIOS 150 hemispherical electron analyzer, and monochromated Al Kα X-rays (hν = 1486.6 eV, Δhν ∼ 0.16 eV) were used as an excitation source. To acquire XPS spectra as a function of RH from 0% to 100% in steps of 10%, a proportional-integral-derivative (PID) controller was employed to regulate the Peltier element so that the Zn sample was maintained at 275 K through feedback provided by the thermocouple, and deionized H_2_O was admitted to the cell at a series of pressures up to 7 mbar; the deionized H_2_O had been thoroughly degassed/cleaned by freeze–pump–thaw cycles before leaking it into the cell.

NAP-XPS data were recorded at normal emission (θ_E_ = 0°), using pass energies of 100 and 40 eV for overview spectra and higher-resolution single core-level spectra, respectively. The C 1s peak at BE = 285 eV was used for binding energy calibration (±0.1 eV). Commercial software, CasaXPS,^49^ was employed for fitting of the higher-resolution spectra, using a combination of line shape functions to describe photoelectron peaks and Shirley-type functions to account for inelastically scattered background electrons.^31^

VSFS data were acquired using a custom-built broadband (BB) system, which was designed to produce mid-infrared (MIR) probe pulses and near-infrared (NIR) pump laser pulses that spatially and temporally coincide at a sample surface, leading to the emission of sum-frequency generation (SFG) photons; the photons were detected by a combination of a Shamrock 163 Czerny–Turner spectrograph (Andor Technology) and an iStar ICCD DH734 intensified-CCD camera (Andor Technology). To produce the two laser pulses, a Ti:sapphire amplifier (Coherent Legend Elite F-HE), seeded by a broadband oscillator (Mai Tai, Spectra Physics), was employed to generate ∼3 mJ pulses with a 120 fs duration, a wavelength centroid of 803 nm (NIR), and a repetition rate of 1 kHz. A portion of this output (1.6 mJ) pumps an Optical Parametric Amplifier (OPerA Solo, Coherent) and is down-converted into tunable MIR pulses at 6 μJ with a duration of ∼120 fs. These pulses are then focused to a spot size of ∼250 μm to comprise the probe beam for the VSFS measurement.

The remainder of the 803 nm light was spectrally narrowed by an air-spaced Fabry–Perot étalon (SLS Optics) and consequently time-stretched to ∼2 ps. This optical element produces a time-asymmetric pulse that allows the nonresonant (nonvibrational) background to be suppressed relative to vibration-induced resonances by the introduction of a time delay (τ) of the NIR pump pulse relative to the MIR probe pulse.^50^ Finally, this 803 nm light was attenuated to ∼12 μJ and focused to a spot size of ∼100 μm. Data presented in this study were acquired using MIR pulses centered at ∼2750 cm^–1^ with the MIR and NIR pulses temporally offset by τ ∼ 730 fs, using a *ppp* polarization combination, i.e., only horizontal p-polarized SFG, MIR, and NIR photons were detected/impinged. Data were also acquired with no temporal offset (τ ∼ 0 fs), but are not shown, as they are entirely dominated by nonresonant background signal.

For the VSFS measurements, the Zn sample was mounted inside a purpose-built sample cell fabricated from polychlorotrifluoroethylene (PCTFE); MIR/NIR laser pulses enter/exit the cell through an uncoated right-angled CaF_2_ prism. To enable data to be acquired as a function of RH (10%–80% in steps of 10%), the sample cell is equipped with ports/tubing to allow air with well-defined RH to flow over the sample. A schematic of this experimental setup is shown in Figure S5. The RH of the incoming air was regulated using a NAFION membrane-based humidifier (Sycos H, Ansyco), which was supplied with RH = 0% air, using a dry air generator (AD41 Dry Air Unit, Oxford Cryosystems). It should be noted that the air was humidified with D_2_O, rather than H_2_O, to minimize intensity losses of the incoming MIR laser pulses due to absorption by the ambient H_2_O vapor in the laboratory. To ensure that any labile −OH species (e.g., OH_ads_ depicted in Figure 1d), were replaced by −OD prior to VSFS measurements, the Zn sample was exposed to air humidified with D_2_O (RH ∼ 80%) for several hours and measurements were initiated once the RH monitor in the postsample glass bottle (see Figure S5) showed RH ≤ 5%.

We note that no unexpected or unusually high safety hazards were encountered during the experimental work.

## Data Availability

Data presented in this paper are available to download from Mendeley Data at https://data.mendeley.com/datasets/jtn7fwxp32.

## Supporting Information Available

Additional NAP-XPS and Auger spectra, XPS and VSFS fitting parameters, details of H_2_O_sor_ coverage calculation, and schematic of VSFS experimental set up (PDF)
