# Supplementary material for: Complexity at a Humid Interface: Throwing Fresh Light on Atmospheric Corrosion
Source: ACS Appl Mater Interfaces. 2025 Apr 23;17(18):27323–30. doi: 10.1021/acsami.4c21013 (PMC12067374; doi:10.1021/acsami.4c21013)
Supplement: Supplementary file 1 — am4c21013_si_001.pdf [file am4c21013_si_001.pdf]

# SUPPORTING INFORMATION

## *Complexity at a Humid Interface: Throwing Fresh Light on Atmospheric Corrosion*

Michael Dowhyj[1,2], Kiran Kousar[1], Francis P. Lydiatt[1,2], Dimitri Chekulaev[1,2], Monika S. Walczak[1], Robert Temperton[3], James N. O'Shea[3], W. Stephen Walters[4], Andrew G. Thomas[2,5], Robert Lindsay[1,2*]

[1]*Corrosion@Manchester, Department of Materials,*

*The University of Manchester, Manchester M13 9PL, UK*

[2]*Photon Science Institute, The University of Manchester, Manchester, M13 9PL, UK*

[3]*School of Physics and Astronomy and, University of Nottingham,*

*Nottingham NG7 2RD, UK*

[4]*UK National Nuclear Laboratory, Culham Science Centre, Abingdon,*

*Oxfordshire, OX14 3DB, UK*

[5]*Department of Materials, The University of Manchester, Manchester M13 9PL, UK*

[*]Corresponding author Email Address:

robert.lindsay@manchester.ac.uk

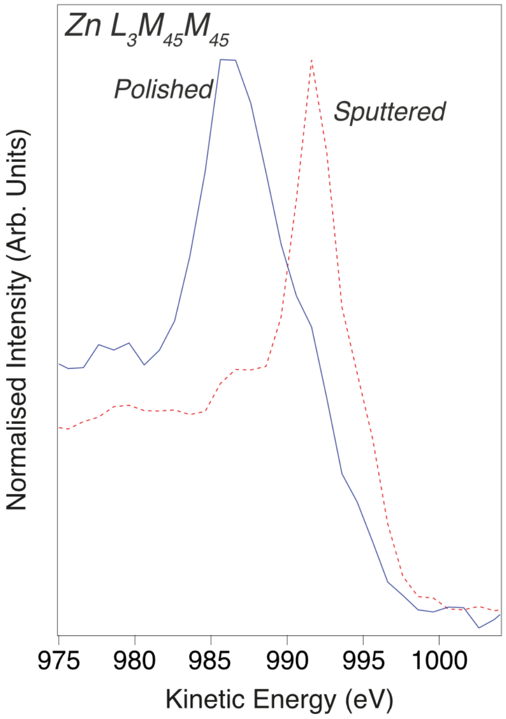


**Figure S1.** X-ray (hν = 1486.6 eV, $\theta_E = 0°$) induced Zn $L_3M_{45}M_{45}$ Auger feature, acquired from a polished Zn substrate that has undergone UV-ozone exposure (solid blue line) and the same surface following $Ar^+$ cluster sputtering to remove surface layers (dashed red line). These data are consistent with previously published spectra of oxidised Zn and metallic Zn,[1] indicating that the polished substrate is terminated by a layer of oxidised Zn. It should be noted that the thickness of oxidised layer is sufficient to minimise signal from the underlying metallic Zn substrate.

**Table S1.** Optimal BEs and FWHMs of the GL(30) line shape functions employed to fit the C 1s and O 1s XPS core level spectra in Fig. 1, which were acquired from a polished/UV-ozone exposed Zn substrate. Component assignments and their labels are also listed, along with references employed for the former.

| BE (eV) | FWHM (eV) | Assignment; Label | Ref. |
|---|---|---|---|
| ***C 1s***[a] | | | |
| 285.0[b] | 1.6 | C in C-C/C-H group in AdC; $C^{C-C/H}$ | 2 |
| 286.6 | 1.6 | C in C-O group in AdC; $C^{C-O}$ | 2 |
| 288.1 | 1.6 | C in C=O group in AdC; $C^{C=O}$ | 2 |
| 289.5[c] | 1.6 | C in O-C=O group in AdC; $C^{O-C=O}$ | 2 |
| ***O 1s*** | | | |
| 530.0 | 1.6 | O in ZnO; $O^{ZnO}$ | 3–6 |
| 531.0 | 1.6 | O in $ZnO_{1-x}$; $O^{ZnO_{1-x}}$ | 7 |
| 531.7[c] | 1.6 | O in $Zn(OH)_2$ + $-OH_{ads}$; $O^{Zn(OH)_2/OH_{ads}}$ | 3–6,8–10 |
| 531.8[c] | 1.6 | O in C=O group in AdC; $O^{C=O}$ | 11,12 |
| 532.6 | 1.6 | O in C-O group in AdC; $O^{C-O}$ | 11,12 |
| 533.6 | 1.6 | O with single bond in O-C=O group in AdC; $O^{O-C=O}$ | 11,12 |
| 535.1 | 1.6 | Shake-up satellite[d]; $O^{sat}$ | 13 |

a. Given that the carbon signal arises from groups present in adsorbed adventitious carbon (AdC), which are difficult to identify with complete certainty, the spectrum was simply fitted with the minimum number of peaks required to achieve an acceptable fit.

b. For each C 1s spectrum, this feature was fixed at BE = 285.0 eV, and the required BE shift was used to calibrate the BE scale of other spectra (e.g., O 1s core level).

c. There may also be a contribution from surface carbonate ($CO_3^{2-}$) to this feature, as such species are readily formed on zinc oxide surfaces. [14]

d. This assignment is based on Ref. [13], where a similar feature (slightly higher BE) appearing in O 1s spectra acquired from $Cu_2O$ was proposed to be a shake-up satellite. Currently, this attribution is speculative, and further work is required to make a robust identification of the origin of this feature.

**Table S2**. Best fit peak areas (pre-normalisation) of the components employed to fit the O 1s XPS core level spectra acquired from a polished/UV-ozone exposed Zn substrate as a function of increasing/decreasing RH. GL(30) line shape functions were employed for peak fitting, except for $H_2O_{(g)}$, where a Gaussian-convoluted Lorentzian based asymmetric line shape function (LA) was used.[15] The BE of each component was constrained to match (± 0.1 eV) those listed in Table S1, and their FWHMs were maintained at 1.5 eV.

| **Component** | **RH(%): INCREASING** | | | | | | | | | | |
|---|---|---|---|---|---|---|---|---|---|---|---|
| | 0 | 10 | 20 | 30 | 40 | 50 | 60 | 70 | 80 | 90 | 100 |
| | Area (CPS eV) | | | | | | | | | | |
| $O^{ZnO}$ | 737.7 | 496.3 | 306.8 | 235.5 | 182.2 | 141.0 | 101.8 | 74.4 | 58.7 | 37.2 | 30.2 |
| $O^{ZnO_{1-x}}$ | 267.7 | 357.1 | 307.3 | 269.6 | 247.7 | 209.6 | 174.3 | 135.4 | 114.6 | 72.6 | 59.0 |
| $O^{Zn(OH)_2/OH_{ads}}$ | 2586.4 | 1569.9 | 879.5 | 637.8 | 456.8 | 335.7 | 219.6 | 151.4 | 112.6 | 71.4 | 57.9 |
| $O^{C=O}$ | 742.6 | 1000.0 | 988.2 | 946.6 | 694.6 | 581.2 | 460.8 | 359.2 | 247.8 | 179.6 | 140.1 |
| $O^{C-O}$ | 573.4 | 232.2 | 252.1 | 134.7 | 136.0 | 86.5 | 91.7 | 106.9 | 82.0 | 74.0 | 49.0 |
| $H_2O_{sor}$ | 0.0 | 277.9 | 285.0 | 280.2 | 250.3 | 211.6 | 161.4 | 118.6 | 97.9 | 79.3 | 95.1 |
| $O^{O-C=O}$ | 317.6 | 259.1 | 131.4 | 125.7 | 82.5 | 83.0 | 93.1 | 89.7 | 89.9 | 79.3 | 63.3 |
| $O^{sat}$ | 150.0 | 135.5 | 115.0 | 80.6 | 97.0 | 78.4 | 63.7 | 37.7 | 28.8 | 28.4 | 23.8 |
| $H_2O_{(g)}$ | 0.0 | 185.9 | 350 | 432.5 | 462.3 | 484.5 | 525.8 | 511.3 | 497.4 | 450 | 433.5 |
| | **RH(%): DECREASING** | | | | | | | | | | |
| | 100 | 90 | 80 | 70 | 60 | 50 | 40 | 30 | 20 | 10 | 0 |
| | Area (CPS eV) | | | | | | | | | | |
| $O^{ZnO}$ | 30.2 | 33.7 | 44.8 | 49.3 | 59.5 | 76.5 | 89.2 | 113.9 | 137.5 | 198.4 | 240.4 |
| $O^{ZnO_{1-x}}$ | 59.0 | 68.0 | 89.0 | 99.9 | 123.0 | 157.3 | 183.0 | 235.0 | 284.7 | 404.0 | 495.2 |
| $O^{Zn(OH)_2/OH_{ads}}$ | 57.9 | 62.3 | 84.2 | 90.4 | 107.0 | 138.6 | 162.2 | 207.0 | 246.7 | 363.2 | 434.3 |
| $O^{C=O}$ | 140.1 | 157.4 | 198.4 | 249.4 | 334.1 | 433.9 | 542.8 | 683.7 | 853.9 | 970.3 | 1223.8 |
| $O^{C-O}$ | 49.0 | 60.2 | 94.9 | 109.4 | 152.0 | 199.3 | 226.6 | 273.0 | 345.7 | 393.9 | 553.9 |
| $H_2O_{sor}$ | 95.1 | 83.0 | 100.9 | 100.4 | 119.6 | 155.3 | 173.2 | 201.8 | 218.9 | 224.3 | 0.0 |
| $O^{O-C=O}$ | 63.3 | 78.1 | 88.7 | 101.2 | 115.9 | 133.0 | 159.9 | 181.0 | 239.6 | 244.5 | 370.8 |
| $O^{sat}$ | 23.8 | 26.1 | 32.2 | 46.9 | 47.8 | 62.0 | 78.4 | 67.0 | 89.1 | 101.2 | 121.0 |
| $H_2O_{(g)}$ | 433.5 | 502.3 | 554.7 | 552 | 554.8 | 598.9 | 547.9 | 495.2 | 409.3 | 214.2 | 0.0 |

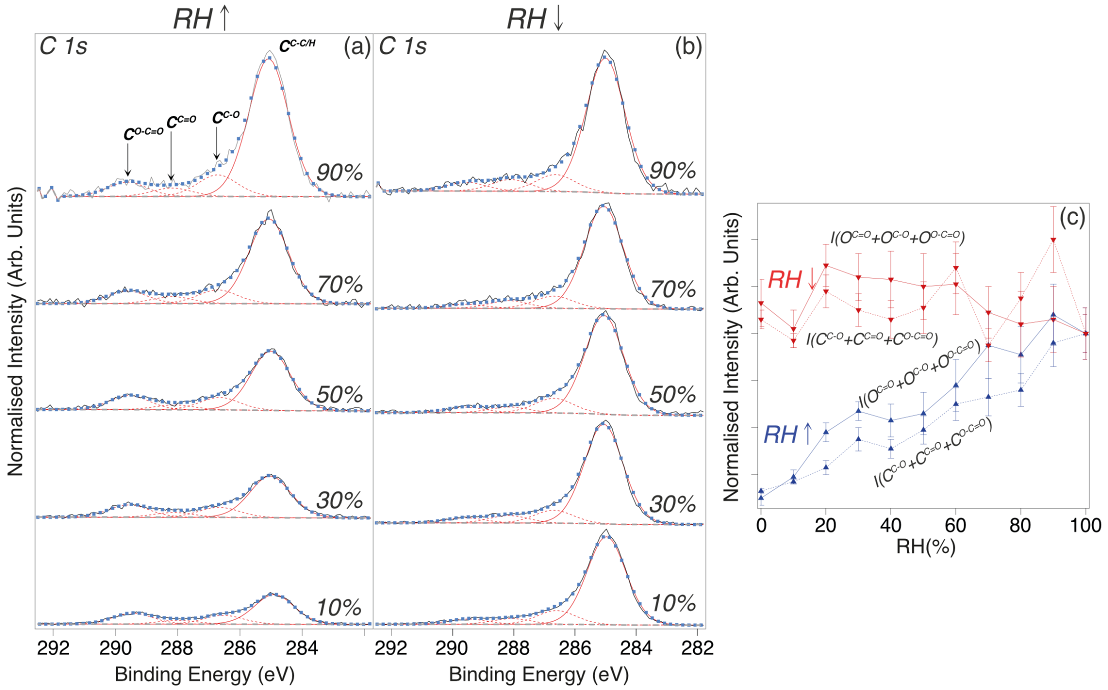


**Figure S2.** C 1s XPS spectra (hν = 1486.6 eV, $\theta_E$ = 0°) acquired from polished/UV-ozone exposed Zn as a function of (a) increasing RH and (b) decreasing RH. Blue markers show best fits to the experimental data (black lines), using GL(30) (red solid/broken lines) line shape functions for all of the components (see Table S3 for best fit peak areas); Shirley-type (broken grey lines) functions were used to describe the secondary electron background.[16] Spectra have been normalised to the sum of the O 1s signals assigned to ZnO, $ZnO_{1-x}$, $Zn(OH)_2/OH_{ads}$ and $O^{sat}$. (c) Plot of similarly normalised summed intensity of $O^{C=O}$, $O^{C-O}$ and $O^{O-C=O}$ ($I(O^{C=O}+O^{C-O}+O^{O-C=O})$, solid line) and $C^{C-O}$, $C^{C=O}$, and $C^{O-C=O}$ (($I(C^{C-O}+C^{C=O}$ +C $C^{O-C=O})$, dashed line) as a function of increasing RH (blue markers) and decreasing RH (red markers). To facilitate a visual comparison, the data have been further normalised to have the same intensity at RH = 100%; error bars have been determined from an estimate of the uncertainty in the best fit.

**Table S3.** Best fit peak areas (pre-normalisation) of the components employed to fit the C 1s XPS core level spectra acquired from a polished Zn substrate as a function of increasing/decreasing RH. GL(30) line shape functions were employed for peak fitting. The BE of each component was constrained to match (± 0.1 eV) those listed in Table S1, and their FWHMs were maintained at 1.5 eV.

| Component | **RH(%): INCREASING** | | | | | | | | | | |
|---|---|---|---|---|---|---|---|---|---|---|---|
| | 0 | 10 | 20 | 30 | 40 | 50 | 60 | 70 | 80 | 90 | 100 |
| | Area (CPS eV) | | | | | | | | | | |
| $C^{C-C/H}$ | 330.1 | 354.5 | 322.5 | 307.8 | 275.3 | 255.5 | 232 | 220.2 | 210.4 | 185.7 | 204.6 |
| $C^{C-O}$ | 160.4 | 106.1 | 77.9 | 74.5 | 58.9 | 53.7 | 51.2 | 35.5 | 29.2 | 29.1 | 24.7 |
| $C^{C=O}$ | 41.5 | 45.2 | 36.0 | 35.7 | 22.7 | 20.9 | 20.3 | 18.1 | 16.2 | 11.3 | 11.6 |
| $C^{O-C=O}$ | 162.9 | 130.2 | 95.9 | 91.5 | 66.9 | 61.0 | 46.4 | 32.8 | 26.2 | 19.3 | 14.8 |
| | **RH(%): DECREASING** | | | | | | | | | | |
| | 100 | 90 | 80 | 70 | 60 | 50 | 40 | 30 | 20 | 10 | 0 |
| | Area (CPS eV) | | | | | | | | | | |
| $C^{C-C/H}$ | 204.6 | 293.4 | 299.2 | 349.2 | 435.9 | 548.8 | 656.6 | 800 | 931.6 | 1100.5 | 1345.8 |
| $C^{C-O}$ | 24.7 | 36.9 | 39.7 | 41.8 | 65.5 | 81.5 | 82.2 | 103.6 | 135.0 | 172.2 | 221.6 |
| $C^{C=O}$ | 11.6 | 25.5 | 24.9 | 26.1 | 30.5 | 32.2 | 46.0 | 59.7 | 68.5 | 70.6 | 106.0 |
| $C^{O-C=O}$ | 14.8 | 19.0 | 21.3 | 13.4 | 32.6 | 30.9 | 34.6 | 41.2 | 63.5 | 65.4 | 83.4 |

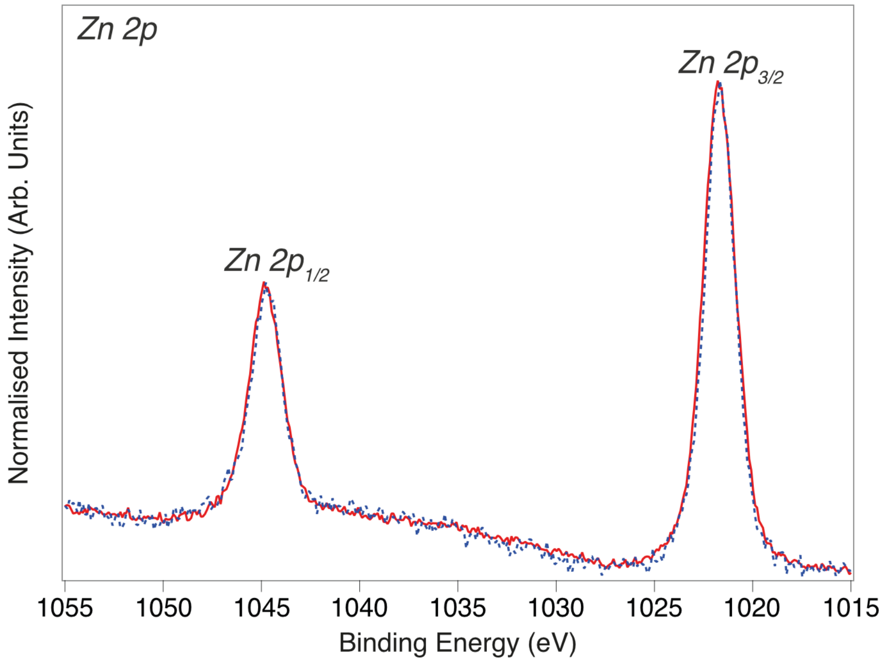


**Figure S3.** Comparison of two Zn 2p XPS spectra, where one was acquired at RH = 10% as RH was increased (solid red line) and the other at the same RH but for decreasing RH (broken blue line). Consistent with the Zn $L_3M_{45}M_{45}$ Auger feature (Figure S1), the BE of the Zn $2p_{3/2}$ peak (1021.8 eV) and the profiles of the spectra, which are essentially identical, correspond to an oxidised Zn surface with no significant contribution from the underlying Zn metal.[1]

**$H_2O_{sor}$ Coverage Calculation**

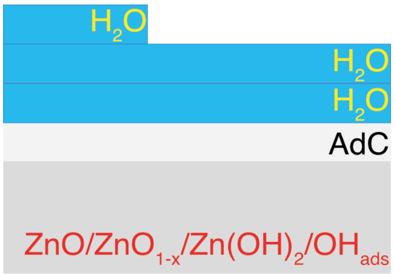


**Figure S4.** Cartoon illustrating surface model assumed for $H_2O_{sor}$ coverage determination.

As illustrated in Figure S4, coverage determination was performed assuming that $H_2O_{sor}$ grows layer-by-layer atop the AdC terminated substrate. In addition, given the Zn $L_3M_{45}M_{45}$ Auger feature profile in Figure S1, it was assumed that metallic Zn does not contribute to the XPS signal, i.e., only the AdC, and $ZnO/ZnO_{1-x}/Zn(OH)_2/OH_{ads}$ layers need to be considered in calculations. On this basis, the following expressions were employed to determine the thickness of the water layer from O 1s component intensities; Table S4 lists values of parameters used to calculate water coverage at RH = 100%).

***For adsorbed water layer ($H_2O_{sor}$):***

$$I_{H_2O} = F \times \sigma_{O1s} \times N_{H_2O} \times K_{O1s} \times \lambda_{H_2O}(E_{O1s}) \times \left\{1 - e^{-t_{H_2O}/\lambda_{H_2O}(E_{O1s})\times cos\theta_E}\right\}$$

$I_{H_2O}$ = Intensity of O 1s signal due to adsorbed water
$F$ = X-ray flux
$\sigma_{O1s}$ = Cross-section for photoionisation of O 1s core level
$N_{H_2O}$ = Number density of the oxygen atoms in adsorbed water
$K_{O1s}$ = Instrumental factor at O 1s KE
$\lambda_{H_2O}(E_{O1s})$ = IMFP for photoelectron in adsorbed water layer at O1s KE
$t_{H_2O}$= Thickness of water layer
$\theta_E$ = Photoelectron emission angle (measured from the sample surface normal)

***For the adventitious carbon layer (AdC):***

$$I_{AdC} = F \times \sigma_{O1s} \times N_{AdC} \times K_{O1s} \times \lambda_{AdC}(E_{O1s}) \times \{[1 - e^{-t_{AdC}/\lambda_{AdC}(E_{O1s})\times cos\theta_E}] \times [e^{-t_{H_2O}/\lambda_{H_2O}(E_{O1s})\times cos\theta_E}]\}.$$

$I_{AdC}$ = Intensity of O 1s signal due to adventitious carbon layer
$N_{AdC}$ = Number density of oxygen atoms in adventitious carbon layer
$\lambda_{AdC}(E_{O1s})$ = IMFP for photoelectron in adventitious carbon layer at O 1s KE
$t_{AdC}$ = Thickness of adventitious carbon layer

***For the ZnO/ZnO$_{1-x}$/Zn(OH)$_2$/OH$_{ads}$ layer:***

$$I_{OZn} = F \times \sigma_{O1s} \times N_{OZn} \times K_{O1s} \times \lambda_{OZn}(E_{O1s}) \times \{[1 - e^{-t_{OZn}/\lambda_{OZn}(E_{O1s})\times cos\theta_E}] \times [e^{-t_{AdC}/\lambda_{AdC}(E_{O1s})\times cos\theta_E}]\} \times [e^{-t_{H_2O}/\lambda_{H_2O}(E_{O1s})\times cos\theta_E}].$$

$I_{OZn}$ = Intensity of O 1s signal due to ZnO/ZnO$_{1-x}$/Zn(OH)$_2$/OH$_{ads}$ layer
$N_{OZn}$ = Number density of O atoms in ZnO/ZnO$_{1-x}$/Zn(OH)$_2$/OH$_{ads}$ layer
$\lambda_{OZn}(E_{O1s})$ = IMFP for photoelectron in ZnO/ZnO$_{1-x}$/Zn(OH)$_2$/OH$_{ads}$ layer at O 1s KE
$t_{OZn}$ = Thickness of ZnO/ZnO$_{1-x}$/Zn(OH)$_2$/OH$_{ads}$ layer

**Table S4.** Values of parameters that were used for calculation of water coverage at RH = 100%.

| Parameter | Value |
|---|---|
| $\theta_E$ | 90° |
| $N_{H_2O}$ [a] | $3.34 \times 10^{22}$ cm$^{-3}$ |
| $N_{AdC}$ [b] | $4.51 \times 10^{22}$ cm$^{-3}$ |
| $N_{OZn}$ [c] | $3.92 \times 10^{22}$ cm$^{-3}$ |
| $\lambda_{H_2O}(E_{O1s})$ [d] | 3.5 nm |
| $\lambda_{AdC}(E_{O1s})$ [e] | 3.0 nm |
| $\lambda_{OZn}(E_{O1s})$ [d] | 2.1 nm |

a. Calculated using density of bulk water at 275 K (ρ = 0.9999 g cm$^{-3}$).[17]
b. Estimated by multiplying number density of carbon atoms in AdC layer by the fraction of those atoms that are bonded to oxygens atoms (extracted from C 1s data in Table5, i.e., I(C$^{C-O}$ + C$^{C=O}$ + C$^{O-C=O}$)/ I(C$^{C-C/H}$ + C$^{C-O}$ + C$^{C=O}$ + C$^{O-C=O}$)), assuming that each these C atoms is bonded to an average 2 O atoms. Following Ref.[18], we assumed that the density of carbon in AdC is equal to that of graphite (ρ = 2.26 g cm$^{-3}$), and so the number density of carbon atoms in adventitious carbon layer is $1.13 \times 10^{23}$ cm$^{-3}$.
c. Estimated from bulk densities of ZnO (ρ = 5.6 g cm$^{-3}$) and Zn(OH)$_2$ (ρ = 3.05 g cm$^{-3}$).[17] Used these values to calculate number density of oxygen atoms in each of these compounds, i.e., $N_{ZnO} = 4.14 \times 10^{22}\ cm^{-3}$ and $N_{Zn(OH)_2} = 3.69 \times 10^{22}\ cm^{-3}$, and adopted mean value.
d. Calculated using Quases-IMFP-TPP2M software.[19] For $\lambda_{OZn}(E_{O1s})$, adopted mean value of IMFPs for ZnO (1.95 nm) and Zn(OH)$_2$ (2.33 nm).
e. Determined using equation (3) in Ref.[20]

***Referring to Ref.*** [21]***, the thickness of the adsorbed water layer ($t_{H_2O}$) can be obtained from the intensity ratio of $I_{H_2O}$ and $I_{OZn}$ through the following expression:***

$$t_{H_2O} = \lambda_{H_2O}(E_{O1s}) \times ln\left(1 + \left(\left(\frac{I_{H_2O}}{I_{OZn}}\right)\left(\frac{N_{OZn}\times\lambda_{OZn}(E_{O1s})}{N_{H_2O}\times\lambda_{H_2O}(E_{O1s})}\right) \times e^{-t_{AdC}/\lambda_{AdC}(E_{O1s})}\right)\right).$$

**$t_{AdC}$ can be obtained from:**

$$t_{AdC} = \lambda_{AdC}(E_{O1s}) \times ln\left(1 + \left(\left(\frac{I_{AdC}}{I_{OZn}}\right)\left(\frac{N_{OZn} \times \lambda_{OZn}(E_{O1s})}{N_{AdC} \times \lambda_{AdC}(E_{O1s})}\right)\right)\right).$$

The thickness of the adsorbed water layer ($t_{H_2O}$) is converted from nm to monolayers (ML) by defining 1 ML of adsorbed water as having the thickness of a molecular layer in bulk liquid water (($N_{H_2O}$) = $3.34 \times 10^{22}$ cm$^{-3}$), i.e., 0.31 nm.

**Fitting of VSFS spectra**

Fitting of the VSFS spectra in Figure 4 was achieved through applying equation (7) in Ref. [22] using a peak fitting routine based on that presented in Ref. [23]. The non-resonant background was fitted to a Gaussian line shape function, and a Voigt line shape function was used to describe the resonant OD stretch ($OD_{free}$) mode. The following fit parameters were employed:

$O_{NR}$ is the offset of the non-resonant background;
$A_{NR}$ is the amplitude of the non-resonant background;
$\nu_{NR}$ is the central wavenumber of the non-resonant background;
$\Delta_{NR}$ is the width of the non-resonant background;

$A_R$ is the amplitude of the resonant mode;
$\nu_R$ is the central wavenumber of the resonant mode;
$\Gamma_R$ is the width of the resonant mode;
$\phi$ is the phase relationship between the resonant mode and the non-resonant background.

**Table S5.** Best fit $A_R$, $\nu_R$, $\Gamma_R$, $\phi$, $O_{NR}$, $A_{NR}$, $\nu_{NR}$, $\phi$, $\Delta_{NR}$ values obtained from fitting of VSFS spectra as a function of RH. Prior to the final fitting, spectra were normalised to the non-resonant background, so that $O_{NR} = 0$ and $A_{NR} = 1$ for all spectra. In addition, $\Gamma_R$, $\Delta_{NR}$, and $\phi$ were constrained to have the same value for all spectra. $\nu_R$ and $\nu_{NR}$ were allowed to vary somewhat to account for both any drift in the central wavelength of the pump and probe beams on the VSFS setup, and for any dispersion caused by the changing humidity in the air.

| | **RH(%): INCREASING** | | | | | | | |
|---|---|---|---|---|---|---|---|---|
| | 10 | 20 | 30 | 40 | 50 | 60 | 70 | 80 |
| | Vibrational resonance | | | | | | | |
| $A_R$ | 0.00 | 4.13 | 5.36 | 7.92 | 8.77 | 8.40 | 10.49 | 10.35 |
| $\nu_R$ | N/A | 2721.1 | 2723.6 | 2724.0 | 2726.0 | 2727.3 | 2726.9 | 2729.8 |
| $\Gamma_R$ | N/A | 5.79 | 5.79 | 5.79 | 5.79 | 5.79 | 5.79 | 5.79 |
| $\phi$ | N/A | 131.77 | 131.77 | 131.77 | 131.77 | 131.77 | 131.77 | 131.77 |
| | Non-resonant background | | | | | | | |
| $O_{NR}$ | 0.0 | 0.0 | 0.0 | 0.0 | 0.0 | 0.0 | 0.0 | 0.0 |
| $A_{NR}$ | 1.0 | 1.0 | 1 | 1.0 | 1.0 | 1.0 | 1.0 | 1.0 |
| $\nu_{NR}$ | 2751.3 | 2740.2 | 2740.6 | 2737.2 | 2731.4 | 2728.5 | 2733.6 | 2729.2 |
| $\Delta_{NR}$ | 140.3 | 140.3 | 140.3 | 140.3 | 140.3 | 140.3 | 140.3 | 140.3 |
| | **RH(%): DECREASING** | | | | | | | |
| | 80 | 70 | 60 | 50 | 40 | 30 | 20 | 10 |
| | Vibrational resonance | | | | | | | |
| $A_R$ | 10.35 | 9.947 | 7.92 | 6.70 | 6.73 | 5.13 | 2.50 | 0.00 |
| $\nu_R$ | 2729.8 | 2728.6 | 2727.1 | 2726.6 | 2725.7 | 2722.7 | 2725.6 | N/A |
| $\Gamma_R$ | 5.79 | 5.79 | 5.79 | 5.79 | 5.79 | 5.79 | 5.79 | N/A |
| $\phi$ | 131.77 | 131.77 | 131.77 | 131.77 | 131.77 | 131.77 | 131.77 | N/A |
| | Non-resonant background | | | | | | | |
| $O_{NR}$ | 0.0 | 0.0 | 0.0 | 0.0 | 0.0 | 0.0 | 0.0 | 0.0 |
| $A_{NR}$ | 1.0 | 1.0 | 1 | 1.0 | 1.0 | 1.0 | 1.0 | 1.0 |
| $\nu_{NR}$ | 2729.2 | 2730.0 | 2729.2 | 2728.8 | 2728.7 | 2732.3 | 2744.6 | 2730.9 |
| $\Delta_{NR}$ | 140.3 | 140.3 | 140.3 | 140.3 | 140.3 | 140.3 | 140.3 | 140.3 |

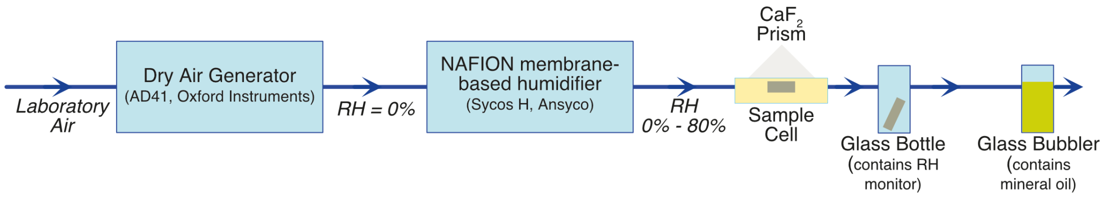


**Figure S5.** Schematic of experimental setup for acquiring VSFS data from a Zn sample as a function of RH (10% – 80%). Air flows from left to right through the various elements, which are connected by PFA tubing. Initially, the air is dried (RH = 0%) using a dry air generator, and then the required RH is achieved with a NAFION membrane-based humidifier. Upon exiting the sample cell, the RH of the flowing air is measured with a RH monitor located in a glass bottle; RH values obtained at this point matched those set with the NAFION membrane-based humidifier. Finally, the air is bubbled through mineral oil, before exhausting into the laboratory.

## References


(1) *Zinc Spectra – ZnO*. https://xpsdatabase.net/zinc-spectra-zno/ (accessed 2024-07-02).

(2) Grey, L. H.; Nie, H.-Y.; Biesinger, M. C. Defining the Nature of Adventitious Carbon and Improving Its Merit as a Charge Correction Reference for XPS. *Appl. Surf. Sci.* **2024**, *653*, 159319.

(3) Kotsis, K.; Staemmler, V. Ab Initio Calculations of the O1s XPS Spectra of ZnO and Zn Oxo Compounds. *Phys. Chem. Chem. Phys.* **2006**, *8* (13), 1490.

(4) Duchoslav, J.; Steinberger, R.; Arndt, M.; Stifter, D. XPS Study of Zinc Hydroxide as a Potential Corrosion Product of Zinc: Rapid X-Ray Induced Conversion into Zinc Oxide. *Corros. Sci.* **2014**, *82*, 356–361.

(5) Winiarski, J.; Tylus, W.; Winiarska, K.; Szczygieł, I.; Szczygieł, B. XPS and FT-IR Characterization of Selected Synthetic Corrosion Products of Zinc Expected in Neutral Environment Containing Chloride Ions. *J. Spectrosc.* **2018**, *2018*, 1–14.

(6) Yu, X.; Roth, J. P.; Wang, J.; Sauter, E.; Nefedov, A.; Heißler, S.; Pacchioni, G.; Wang, Y.; Wöll, C. Chemical Reactivity of Supported ZnO Clusters: Undercoordinated Zinc and Oxygen Atoms as Active Sites. *ChemPhysChem* **2020**, *21* (23), 2553–2564.

(7) Kwoka, M.; Kulis-Kapuscinska, A.; Zappa, D.; Comini, E.; Szuber, J. Novel Insight on the Local Surface Properties of ZnO Nanowires. *Nanotechnology* **2020**, *31* (46), 465705.

(8) Gautier, P.; Vallee, A.; Sinito, C.; Etcheberry, A.; Simon, N. Effect of Growth Temperature on the Electrodeposition of Zinc Oxide Layers on Diamond Surfaces. *Diam. Relat. Mater.* **2016**, *62*, 1–6.

(9) Newberg, J. T.; Goodwin, C.; Arble, C.; Khalifa, Y.; Boscoboinik, J. A.; Rani, S. ZnO($10\bar{1}0$) Surface Hydroxylation under Ambient Water Vapor. *J. Phys. Chem. B* **2018**, *122* (2), 472–478.

(10) Yu, X.; Schwarz, P.; Nefedov, A.; Meyer, B.; Wang, Y.; Wçll, C. Structural Evolution of Water on ZnO(101¯ 0): From Isolated Monomers via Anisotropic H-Bonded 2D and 3D Structures to Isotropic Multilayers. *Angew. Chem. Int. Ed.* **2019**, *58*, 17751-17757.

(11) Briggs, David.; Beamson, Graham. XPS Studies of the Oxygen 1s and 2s Levels in a Wide Range of Functional Polymers. *Anal. Chem.* **1993**, *65* (11), 1517–1523.

(12) Kousar, K.; Walczak, M. S.; Ljungdahl, T.; Wetzel, A.; Oskarsson, H.; Restuccia, P.; Ahmad, E. A.; Harrison, N. M.; Lindsay, R. Corrosion Inhibition of Carbon Steel in Hydrochloric Acid: Elucidating the Performance of an Imidazoline-Based Surfactant. *Corros. Sci.* **2021**, *180*, 109195.

(13) Trotochaud, L.; Head, A. R.; Pletincx, S.; Karslıoğlu, O.; Yu, Y.; Waldner, A.; Kyhl, L.; Hauffman, T.; Terryn, H.; Eichhorn, B.; Bluhm, H. Water Adsorption and Dissociation on Polycrystalline Copper Oxides: Effects of Environmental Contamination and Experimental Protocol. *J. Phys. Chem. B* **2018**, *122* (2), 1000–1008.

(14) Koitaya, T.; Yamamoto, S.; Shiozawa, Y.; Yoshikura, Y.; Hasegawa, M.; Tang, J.; Takeuchi, K.; Mukai, K.; Yoshimoto, S.; Matsuda, I.; Yoshinobu, J. $CO_2$ Activation and Reaction on Zn-Deposited Cu Surfaces Studied by Ambient-Pressure X-Ray Photoelectron Spectroscopy. *ACS Catal.* **2019**, *9* (5), 4539–4550.

(15) Fairley, N. *CasaXPS Manual 2.3.15 Spectroscopy 1.3*; Casa Software Ltd, 2009.

(16) Shirley, D. A. High-Resolution X-Ray Photoemission Spectrum of the Valence Bands of Gold. *Phys. Rev. B* **1972**, *5* (12), 4709–4714.

(17) CRC Handbook of Chemistry and Physics, 85th Ed.; *Lide, D.R., Ed.; CRC Press: Boca Raton, FL, 2004*, 85th ed.; CRC Press.

(18) Comini, N.; Huthwelker, T.; Diulus, J. T.; Osterwalder, J.; Novotny, Z. Factors Influencing Surface Carbon Contamination in Ambient-Pressure x-Ray Photoelectron Spectroscopy Experiments. *J. Vac. Sci. Technol. A* **2021**, *39* (4), 043203.

(19) *QUASES-IMFP-TPP2M Vers. 3.* http://www.quases.com/products/quases-imfp-tpp2m/ (accessed 2024-11-12).
(20) Smith, G. C. Evaluation of a Simple Correction for the Hydrocarbon Contamination Layer in Quantitative Surface Analysis by XPS. *J. Electron Spectros. Relat. Phenomena* **2005**, *148* (1), 21–28.
(21) Yamamoto, S.; Kendelewicz, T.; Newberg, J. T.; Ketteler, G.; Starr, D. E.; Mysak, E. R.; Andersson, K. J.; Ogasawara, H.; Bluhm, H.; Salmeron, M.; Brown, G. E.; Nilsson, A. Water Adsorption on α-$Fe_2O_3$(0001) at near Ambient Conditions. *J. Phys. Chem. C* **2010**, *114* (5), 2256–2266.
(22) Bain, C. D.; Davies, P. B.; Ong, T. H.; Ward, R. N.; Brown, M. A. Quantitative Analysis of Monolayer Composition by Sum-Frequency Vibrational Spectroscopy. *Langmuir* **1991**, *7* (8), 1563–1566.
(23) Hommel, E.L. A Broad Band Sum Frequency Generation Spectroscopic Investigation of Organic Liquid Surfaces, Ohio State University, Ohio, USA, 2003.
